# The Resting Human Brain and Motor Learning

**DOI:** 10.1016/j.cub.2009.04.028

**Published:** 2009-06-23

**Authors:** Neil B. Albert, Edwin M. Robertson, R. Chris Miall

**Affiliations:** 1 Behavioural & Brain Sciences Centre, School of Psychology, University of Birmingham, Birmingham B155 2TT, UK; 2 Department of Psychology, University of Chicago, 5848 S. University Ave., Green Hall 317, Chicago, IL 60637, USA; 3 Berenson-Allen Center for Non-Invasive Brain Stimulation, Harvard Medical School, Beth Israel Deaconess Medical Center, 330 Brookline Ave., Kirstein Building KS 221, Boston, MA 02215, USA

**Keywords:** SYSNEURO

## Abstract

Functionally related brain networks are engaged even in the absence of an overt behavior. The role of this resting state activity, evident as low-frequency fluctuations of BOLD (see [1] for review, [2–4]) or electrical [5, 6] signals, is unclear. Two major proposals are that resting state activity supports introspective thought or supports responses to future events [7]. An alternative perspective is that the resting brain actively and selectively processes previous experiences [8]. Here we show that motor learning can modulate subsequent activity within resting networks. BOLD signal was recorded during rest periods before and after an 11 min visuomotor training session. Motor learning but not motor performance modulated a fronto-parietal resting state network (RSN). Along with the fronto-parietal network, a cerebellar network not previously reported as an RSN was also specifically altered by learning. Both of these networks are engaged during learning of similar visuomotor tasks [9–22]. Thus, we provide the first description of the modulation of specific RSNs by prior learning—but not by prior performance—revealing a novel connection between the neuroplastic mechanisms of learning and resting state activity. Our approach may provide a powerful tool for exploration of the systems involved in memory consolidation.

## Results and Discussion

### Motor Performance and Motor Learning

**Figure 1:**
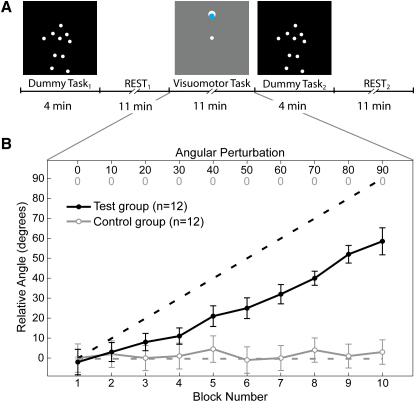
Experimental Design and Performance during the Visuomotor Task (A) The experiment began with a dummy task and a baseline rest condition (REST_1_, 11 min) followed by the visuomotor task (11 min). Then participants completed a second dummy task before the final rest condition (REST_2_, 11 min). The dummy task display was of point light displays of human whole-body movements, or scrambled versions that showed the same individual dot motions, but with random positions. The visuomotor task display shows the central start location, a target and the cursor. (B) In the visuomotor task the relative angle of the cursor motion compared to the joystick gradually increased with each block, for the test group (dashed group), but remained veridical for the control group. The mean direction of joystick movement with respect to the target (solid line, ±1 SEM) steadily increased for the test group (black) and remained constant for the control group (gray).

To measure the modulation of resting state activity after a short period of sensorimotor learning, we exposed two groups of participants to one of two versions of a visuomotor “center-out” tracking task [23] (Figure 1A; see Supplemental Experimental Procedures available online). The test group (n = 12) adapted their joystick movements to a novel relationship between cursor and joystick (motor learning), whereas the control group (n = 12) performed similar tracking movements but with veridical cursor feedback of the joystick (motor performance).

In the test group, the movement of the cursor relative to the joystick was gradually rotated about the center of the screen, increasing by 10° each minute (dashed line, Figure 1B). Thus both groups began the task with 0° perturbation and their performance was initially comparable (see Supplemental Results, Behavioral Results). But during the remaining 10 min, the movements of the test group clearly reflected their progressive compensation for the visuomotor perturbation. By the end of the visuomotor task, the mean joystick direction for the test group was rotated by 58.7° with respect to the target direction (black line, Figure 1B). This level of adaptation, compensating for 65% of the imposed perturbation, is similar to performance observed in other experiments (see also Supplemental Experimental Procedures, Behavioral Protocols) (e.g., [24, 25]).

### Model-Free Whole-Brain Probabilistic Independent Components Analysis

Probabilistic independent components analysis (PICA) of the BOLD signal allowed us to identify the networks evident during rest [26] and to measure changes in these components after motor learning (test group, n = 12) or motor performance (control group, n = 12). We contrasted the engagement of these networks identified by PICA before (REST_1_) and after (REST_2_) the visuomotor task. To ensure that the second resting period was not affected by perseverating on the motor task, we preceded each rest period by a 4 min “dummy” task, in which the subjects observed point light displays of human movements or scrambled dots (Figure 1A; see Experimental Procedures for details).

#### Baseline Analysis

**Figure 2:**
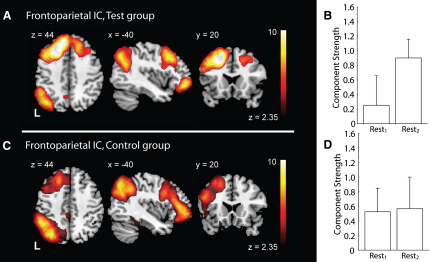
A Fronto-Parietal Resting State Network that Increased in Strength after Exposure to the Visuomotor Adaptation, but Not Performance This independent component was identified as reliable across the participants in each group and across both rest blocks. The fronto-parietal network (A, C) closely corresponds to a previously identified RSN [3, 4]. The strength of the fronto-parietal network during rest was increased after motor learning (B), but not after motor performance (D).

To first check comparable baseline activity in the two groups, REST_1_ data for both groups were combined in a single PICA analysis with a between-groups contrast. This concatenation of data across participants allows the PICA analysis to identify spatially consistent regions across the groups that are correlated in their BOLD signal activity, but without the constraint that the activity in individual participants is temporally correlated with other participants or with any external stimulus time course [26]. We identified six previously reported RSNs (see Figures 2A–2E and 2H of [4]). None of these components significantly varied between groups during the initial resting session (each t(22) < 0.56, each p > 0.29).

#### Analysis of Learning-Dependent Change

**Figure 3:**
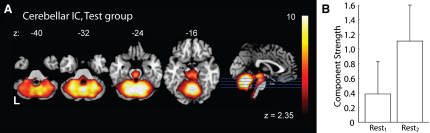
Resting State Activity within the Cerebellum Increased in Strength after Exposure to the Visuomotor Adaptation Task This independent component (A) was reliably identified across the combined data for both rest sessions in the test group across, and significantly differed between the two rests (B). The absence of this network in previous reports on resting state networks and its absence in the control group suggests that activation of this network may have been driven by the motor learning experience.

The BOLD data from both sessions (REST_1_ and REST_2_) were then analyzed for each group (test and control) independently, testing for RSN components that changed in strength after motor learning (in the test group) or motor performance (in the control group). In the test group, a fronto-parietal (Figure 2) and a cerebellar (Figure 3) component were reliably identified across both REST sessions and significantly increased in strength after motor learning. In the control group, the fronto-parietal component (but not the cerebellar component) was reliably identified in both rest sessions, and this component did not change in strength after the visuomotor task. This increase in component strength reflects an increase in the BOLD signal variability that can be attributed to a particular component.

The fronto-parietal component included the prefrontal cortex, the superior and inferior parietal cortex, and Crus II of the cerebellum (see Table S1). This component was reliable across both rest sessions in the test group (z = 1.91, p = 0.028; Figure 2A) and across both rest sessions in the control group (z = 1.65, p = 0.01; Figure 2C), but only changed from REST_1_ to REST_2_ in the test group (i.e., after motor learning; t(11) = 2.074, p = 0.031; Figure 2B). The fronto-parietal component had also been reliably identified in our baseline analysis comparing REST_1_ data between the two groups (Figure S1A; z = 2.28, p = 0.01), and its baseline activity was not significantly different between groups (Figure S1B; t(22) = −0.42, p = 0.34). Thus, the fronto-parietal component, though similar in both groups during the initial resting scan, was altered only after learning.

Additionally, a component that encompassed the majority of the cerebellum was identified in the analysis across both rest sessions in the test group (Figure 3A; z = 1.78, p = 0.038), and this component also significantly increased after learning the novel motor skill (t(11) = 1.880, p = 0.043; Figure 3B). This component had not been identified in our combined baseline (i.e., test and control group) analysis of REST_1_, however, suggesting that it may be qualitatively different from conventional RSNs. No other components were identified by the PICA analysis that significantly increased or decreased in strength between REST_1_ and REST_2_.

The ICA approach identifies regions with correlated patterns of resting activity. To explore whether the learning-dependent changes we identified have additional, within-component structure, we additionally performed within-subject, within-session whole-brain correlations against the time-course of BOLD signal recorded within small “seed” regions of interest (see Table S1). The 48 resulting covariance maps for each seed ROI (2 groups of 12 subjects, two sessions) were then tested for significant group × session interactions. Detailed description is beyond the scope of this short report, but we found significant group × session interactions between (1) inferior frontal gyrus, middle frontal gyrus, and cerebellar lobule IX, (2) superior frontal gyrus and fusiform cortex, (3) the angular gyrus and hippocampus, and (4) the precentral gyrus and the middle frontal gyrus and inferior frontal cortex (see Supplemental Results). Thus the main group × session interactions are within the components identified by the PICA analysis; however, there are small but significant regions lying outside of the fronto-parietal and cerebellar components that are affected by motor learning.

Our results demonstrate that motor learning, but not motor performance, modulates subsequent resting activity in specific task-relevant networks. The fronto-parietal network was identified in both groups within their initial resting brain activity (see Figure S1) but was modulated in the test group only after the acquisition of a novel motor skill (see Figure 2). In contrast, when there was no motor skill to learn (i.e., in the control group), there was no change in the spontaneous activity after motor performance. Thus, neuroplastic changes, driven by learning a novel motor skill, shaped subsequent spontaneous activity within the resting brain. This demonstrates a link between neuroplastic processing and resting brain activation, which has implications for both our understanding of memory processing and the functional interpretation of resting brain activity.

Changes in resting state activity were induced specifically by learning. The tasks performed by the two groups were virtually identical, with the exception that the test group learned to compensate for gradually shifting visuomotor feedback. We found no evidence of any change in movement direction, peak velocity, or latency in the control group, and the performance measure of interest—the direction of their joystick motion—was stable throughout. Accordingly, the significant changes observed in the two resting state components in the test group (Figures 2 and 3) are attributable to learning. This is an important distinction from an earlier report of offline persistence of memory-related activity [27]. That work was not able to test whether the activity measured in an auditory odd-ball task, modulated by exposure to one of two different learning tasks, was influenced by task performance or by learning.

Changes in resting activity were not limited to the time immediately after learning, but were measured after conscious processing has been redirected to an unrelated dummy task for a period of 4 min. Consequently, our results should not be confounded by processing attributable to ruminating about the tracking task. This is a critical feature of the data reported here, because the persistence of neural activity across unrelated tasks would be necessary of any process that could lead to memory consolidation, which takes place over several hours (or overnight) after exposure to learning [28].

The networks affected by visuomotor adaptation, including the fronto-parietal (Figure 2) and cerebellar circuits (Figure 3), are known to be active during visuomotor adaptation [14, 15, 18–21] and are necessary for the long-term retention of motor skills [16, 17, 22]. In fact, there is a striking overlap between the areas identified with PICA in this experiment and areas involved in motor learning (see [29] for review) and areas that represent consolidated motor skills (see [30] for review).

Because a global cerebellar RSN has not been previously reported and because this component was not identified across the two groups during the baseline REST_1_ session, it is important to scrutinize this result in greater detail. It may be the case that the learning task for the test group so strongly engaged this network in REST_2_ (Figure 3B) that its increased strength after learning significantly contributed to the overall variability across *both* rest sessions. Hence we suggest that it has been identified only in the test group data because of its activation by learning. Previous imaging reports suggest widespread cerebellar activation during active performance of motor learning tasks [10, 12, 17], but as far as we are aware, no others have searched for cerebellar resting state components after a period of motor learning. In other words, global engagement of the cerebellum may not be typical during rest. Rather, its engagement may require recent cerebellum-dependent learning and its engagement would not be expected without such learning.

Activity within the resting brain may reflect the on-going “off-line” processing of information gained from earlier learning [8, 27, 31]. Short-term memories for past experiences are consolidated over time [31–35] and the processing and metabolic demands of consolidation must be met by the resting brain [8]. It is possible that these processes might also be reflected in the slow fluctuations of BOLD signal that are detected as RSNs. Moreover, consolidation processes would be expected to modulate the strength of cortico-cortical interactions [36], and thus be evident as the increase in strength of spatio-temporal patterns identified by PICA analysis. Thus, strengthening of PICA components, which indicates an increase in the proportion of BOLD signal variability explained by that component, may reflect greater correlated activity within the brain areas comprising the component. This was confirmed by correlational analysis briefly described above (see Supplemental Results) suggesting localized changes within these networks that will require additional research.

In conclusion, we have shown that motor learning, but not motor performance, can modulate particular resting state networks. This reveals a novel connection between neuroplasticity and subsequent resting state activity, which may in part arise because the off-line processing of memory during consolidation is supported by task-specific resting state activity. Our results add a new dimension to our understanding of the resting brain and potentially provide a powerful new technique to examine the neuronal machinery of off-line processing.

## Experimental Procedures

### Participants

We recorded BOLD signal from 24 right-handed participants over five consecutive conditions within a single scanning session (Figure 1A; see Supplemental Experimental Procedures for full details). Participants were randomly assigned to either the test (6 men and 6 women; age: mean = 27.0 years, SEM = 2.77 years) or the control (5 men and 7 women; age: mean = 24.6 years, SEM = 1.39 years) group. Informed consent was obtained from each participant, and the experiment was approved by our local ethical committee. Participants received financial compensation for their time.

### Behavioral Protocol

A 4 min dummy task immediately preceded each rest session, in which the participant passively viewed dynamic point light displays of human whole-body movements or scrambled versions that showed the same individual dot motions, but with random positions [37]. Individual stimuli lasted 3 s and were blocked into 30 s interleaved runs of 10 human and 10 scrambled motion stimuli. The participant was instructed to attend to the stimuli, discriminating human and scrambled movements, but had no active task to perform.

The visuomotor task [23] (see Supplemental Experimental Procedures) interleaved between the two rest sessions required the participants to use their nonpreferred left hand to move an MR-compatible joystick. In the test group, there was a novel angular displacement of 10° between the cursor and joystick position introduced every minute over 10 min, which produced a final 90° displacement. In the control group there was no novel relationship between the cursor and joystick position. Tracking performance was assessed in both groups by calculating the direction of the joystick with respect to the target during the first 100 ms of each movement, averaged across each block of 24 movements.

### fMRI Analysis

Resting state analysis was carried out with PICA [26] as implemented by MELODIC (Multivariate Exploratory Linear Decomposition into Independent Components) Version 3.05, which is a part of FSL (Functional Magnetic Resonance Imaging of the Brain Software Library, http://www.fmrib.ox.ac.uk/fsl). Correlational analysis was performed with a GLM model within FEAT (FMRI Expert Analysis Tool, also within the FSL package). See Supplemental Experimental Procedures for further details.

## Supplemental Data

Document S1. Supplemental Results, Supplemental Experimental Procedures, Three Figures, and Three Tables
