# Supplementary material for: The Resting Human Brain and Motor Learning
Source: Curr Biol. 2009 Jun 23;19(12):1023–7. doi: 10.1016/j.cub.2009.04.028 (PMC2701987; doi:10.1016/j.cub.2009.04.028)
Supplement: Document S1. Supplemental Results, Supplemental Experimental Procedures, Three Figures, and Three Tables [file mmc1.pdf]

Current Biology, Volume *19*

Supplemental Data

The Resting Human Brain and Motor Learning

Neil B. Albert, Edwin M. Robertson, and R. Chris Miall

Supplementary Results

Behavioral Results

We assessed two additional features of the tracking movements, to test for non-specific changes in performance: the peak velocity of each outward movement and the latency of this moment from the onset of the target. The test group reached lower peak velocities (mean ± SEM: test = 2.16 ± 0.8°/s, control = 4.30 ± 0.8°/s; $F(1,20) = 368.12$, $p < 0.001$), but these occurred at a similar latency from the target onset in both groups (mean ± SEM: test = 731 ± 25ms, control = 701 ± 23ms; $F(1,20) < 1$). Critically, neither peak velocity nor its latency varied across the tracking session for either test or control groups (Group × Block interactions: $F(9,180) < 1$ in each case). In addition, the average directional errors of the control group were small and stable across the whole block (Figure 1B, main paper, grey solid line). Thus, the only indication of learning was in the initial direction of the joystick movements produced by individuals within the test group.

FMRI Results

Independent Components Analyses

To confirm that the component identified as modulated by learning (Figure 2, main paper) was also reliably identified in the pre-learning rest session, we concatenated the $REST_1$ data from the two participant groups into a single analysis. We identified a component (Supplementary Figure 1) that was very similar to the fronto-parietal component that was

modulated by motor learning in the test group (compare with Figure 2, main paper). The strength of this component was not significantly different between the two groups ($t(22) = 0.42$, $p = 0.68$). Thus this component was present in both groups initially, but was only affected by the visuo-motor task in the learning group.

Correlational Analyses

To verify our ICA analysis, we used ROI-based correlation analysis to calculate a mean covariance map for the $REST_1$ session across both groups (equivalent to the data shown in Supplementary Figure 1). A 5-mm region of interest was located in left superior frontal gyrus (xyz: -20, 26, 48), based on the local maximum coordinates in Supplementary Table 1. The correlation between BOLD signal in this ROI and all other voxels was calculated using a GLM analysis. As expected, the regions identified (Supplementary Figure 2) were close to those seen in the independent component analysis.

We then performed a 2x2 mixed effects ANOVA on correlational analyses for 5 seed ROIs, with group (test and control) and session ($REST_1$ vs $REST_2$) as factors. Significant positive or negative interactions were identified with uncorrected threshold of $p=0.001$ (Supplementary Table 3). Supplementary Table 3 indicates areas where the strength of correlation with these ROIS was significantly modulated by learning, as identified by significant interaction between the group (test vs control) and session ($REST_1$ vs $REST_2$) factors. Notable was a negative interaction between the left angular gyrus (xyz: -46, -70, 44) and the left hippocampus (Supplementary Figure 2) and positive interactions between left precentral gyrus (xyz: -42, 12, 44) and left middle frontal gyrus (BA45, Supplementary Figure 3A) and left inferior frontal cortex (BA47; Supplementary Figure 3B). These results confirm that the areas in which the correlation with the target region was significantly modulated by learning are largely

confined within the component identified by ICA, but also suggest that there is a complex intra-component network of correlations that will require detailed analyses to fully understand.

BOLD-behavior correlations

The change in strength of the two RSN components identified by PICA across participants within the test group (Figure 2, main paper) was not linearly correlated with behavioral measures of learning, but this does not imply there is no relationship. Our task instructions emphasized movement direction, rather than performance speed or terminal accuracy and so several different indices of learning might interact in defining the overall pattern of change in resting state activity [7, 8]. The gradual increase in the visuo-motor perturbation throughout the task was chosen to maximize adaptation to the task, but did not allow a clear measure of improved and retained skill. Additionally, there are between-subjects differences in baseline competence with our joystick, so we expect differences in learning rates across the group that may have no simple linear relationship with consolidation-related processing. Further investigation with much greater sample sizes and with assessments of individual differences before and after a training session will be necessary to fully address the quantitative relationship between behavioral measures of learning and changes in the resting brain.

Supplemental Experimental Procedures

Behavioral protocols

Participants were scanned throughout 5 consecutive sessions (Figure 1, main paper) taking a total of 45 minutes. The first was a 4 minute dummy task designed to ensure a common cognitive baseline, which immediately preceded each rest session. The participant passively viewed dynamic point light displays of human whole body movements, or scrambled versions that showed the same individual dot motions, but with random positions [1]. Individual stimuli

lasted 3s and were blocked into 30s interleaved runs of 10 human and 10 scrambled motion stimuli. The participant was instructed to attend to the stimuli, discriminating human and scrambled movements, but had no active task to perform.

The dummy task was followed by an 11-minute rest session, in which the participant was instructed to remain relaxed, with eyes closed. This was then followed by the visuo-motor task. Participants held the joystick case with their right hand and used their left hand to make small controlled movements of the joystick. Movements of the joystick tip of 1cm produced a 5.5cm on-screen cursor movement. Initially, visual feedback was veridical so that movement of the joystick towards the participant's feet elicited an upward movement of the cursor on the screen; left and right movements were veridical. A target appeared every 800 ms at one of 8 positions on a circle circumference centered on the start position, in pseudorandom order. After each 30 seconds (24 movements), target and cursor color changes cued participants to passively view the presented targets for 30 seconds. At the onset of the each successive active tracking block, in the test group the angular relationship between the joystick and cursor movement increased by 10° clockwise. Thus, the increasing visuomotor perturbation required test group participants to move the joystick counter-clockwise to the presented target on the screen, in order to direct the cursor towards the target. The cursor rotation increased by 10° each minute, throughout the 11 minute tracking task. For technical reasons, tracking data from the final block was lost for several participants. We therefore report tracking performance for only the first 10 blocks when the angular displacement in the test group had reached 90 degrees. Upon completion of the experiment, all participants expressed awareness of the existence of a visuo-motor perturbation.

Participants in the control group completed a very similar task to that described above. The only difference was that the angular relationship between the joystick and cursor movement

remained veridical throughout the 11 minute tracking task. An additional control group (n=14) completed the same adaptive task as the test group, but in the laboratory, and were then tested during the reintroduction of the veridical environment after the final adaptation block. This group showed the same level of adaptation as the test group, and also showed an aftereffect of 22° when returned to the veridical, unrotated condition, confirming learning.

The visuo-motor session was followed by another 4-minute dummy-task session, identical to the first, and was immediately followed by the second resting session, again identical to the first session. To additionally control for differences in mental state between the two rest sessions, other than learning, participants in both groups were falsely instructed that they would complete a second session of the tracking task after the second rest period. Thus both rest sessions were preceded by the same dummy task, and were undertaken in the expectation of a subsequent tracking task.

FMRI Acquisition.

218 T2*-weighted echo planar images (EPIs) were acquired using a 3T Philips Achieva scanner (Koninklijke Philips Electronics N.V., Eindhoven, Netherlands) during the resting and visuomotor blocks (TR =3 000ms; TE = 35ms; flip angle = 85°) using a SENSE head coil (SENSE factor 2). Each EPI volume was comprised of 49 96×96 axial slices of 2.5mm × 2.5mm × 3mm voxels, which covered the entire cerebral cortex and cerebellum (FOV = 240mm × 147mm × 240mm). A high-resolution T1-weighted structural volume (TR = 8.4ms; TE = 3.8ms; flip angle = 8°, FOV = 232mm × 288mm × 175mm) was also acquired for use during coregistration and normalization of the EPIs to the ICBM152-template [2] resliced to 2mm thick slices.

Independent Components Analyses

Independent analyses were run on each group, and the following procedures were followed for each of those analyses. The 24 EPI rest scans (218 volumes each) were concatenated in the model, along with a contrast model dissociating $REST_1$ sessions from $REST_2$ sessions. Thus, the PICA analysis would identify spatially consistent components across the 24 scans, without requiring common temporal structure. Each EPI volume was motion-corrected using MCFLIRT [3], high-pass filtered (0.01HZ cutoff), masked to eliminate non-brain voxels, spatially-smoothed using a 5mm FWHM filter, demeaned on a voxel-by-voxel basis, whitened, and projected into a 48-dimensional subspace using PICA. The dimensionality of the subspace was estimated using the Laplace approximation to the Bayesian evidence of the model order [4] for the test group, and set to 48 (the value from the approximation in the test group) for the control group. Non-brain structures were removed from the high-resolution structural image using BET [5] and the transformation matrix used for the affine registration of this image to the ICBM152 brain [2] was applied to the PICA output from each session.

The whitened observations were decomposed into sets of vectors which describe signal variation in the temporal domain (time-courses) across the spatial domain (maps) by optimizing for non-Gaussian spatial source distributions using a fixed-point iteration technique [6]. Estimated component maps once derived were used to generate an estimate of the error variance, which was used to convert the individual component maps into Z-score maps. These maps were then converted into probabilistic component maps by fitting the individual Z-score maps with Gamma/Gaussian Mixture-Models [4]. Components identified as reliably non-zero across the 24 scans were visually inspected to ensure that they were spatially similar to previously identified

resting networks, were not heavily influenced by any single scan, and contained limited power in frequencies above 0.1Hz. Each remaining component was tested using an ordinary least squares general linear model to find those that significantly differed in strength between the two REST sessions and were reliably non-zero across participants.

Correlational Analyses

Regions of interest were chosen based on the coordinates of local maxima within the main significant impendent component identified within fronto-parietal cortex (Figure 1, main paper). A 5mm radius spherical region was centered on each of 5 coordinates (see Table 1), and transformed into the original image space for each individual recording session (24 participants, 2 sessions). The mean BOLD signal within the ROI was then calculated from the preprocessed and filtered 4-D dataset for each data set. This temporal signal was used as a covariate for a whole-brain GLM analysis, in order to calculate the whole-brain covariance with the seed region. The 48 maps calculated for each of the 5 seed regions were then compared in a 2x2 mixed design, testing for significant group×session interactions. Positive interaction would identify areas where the correlation with the seed region was selectively enhanced after learning, whereas negative interactions would identify areas where there was a selective reduction in correlation,

References

1. Jastorff, J., Kourtzi, Z., and Giese, M.A. (2006). Learning to discriminate complex movements: biological versus artificial trajectories. Journal of vision 6, 791-804.
2. Mazziotta, J., Toga, A., Evans, A., Fox, P., Lancaster, J., Zilles, K., Woods, R., Paus, T., Simpson, G., Pike, B., et al. (2001). A probabilistic atlas and reference system for the human brain: International Consortium for Brain Mapping (ICBM). Philos. Trans. R. Soc. Lond. B. Biol. Sci. 356, 1293-1322.
3. Jenkinson, M., Bannister, P., Brady, M., and Smith, S. (2002). Improved optimization for the robust and accurate linear registration and motion correction of brain images. NeuroImage 17, 825-841.
4. Beckmann, C.F., and Smith, S.M. (2004). Probabilistic independent component analysis for functional magnetic resonance imaging. IEEE Trans. Med. Imaging 23, 137-152.
5. Smith, S.M. (2002). Fast robust automated brain extraction. Hum. Brain Mapp. 17, 143-155.
6. Hyvarinen, A. (1999). Fast and robust fixed-point algorithms for independent component analysis. IEEE Trans. Neural Networks 10, 626-634.
7. Diedrichsen, J., Hashambhoy, Y., Rane, T., and Shadmehr, R. (2005). Neural correlates of reach errors. J. Neurosci. 25, 9919-9931.
8. Hikosaka, O., Nakahara, H., Rand, M.K., Sakai, K., Lu, X., Nakamura, K., Miyachi, S., and Doya, K. (1999). Parallel neural networks for learning sequential procedures. Trends Neurosci. 22, 464-471.
9. Tzourio-Mazoyer, N., Landeau, B., Papathanassiou, D., Crivello, F., Etard, O., Delcroix, N., Mazoyer, B., and Joliot, M. (2002). Automated anatomical labeling of activations in SPM using a macroscopic anatomical parcellation of the MNI MRI single-subject brain. NeuroImage 15, 273-289.
10. Raichle, M.E., MacLeod, A.M., Snyder, A.Z., Powers, W.J., Gusnard, D.A., and Shulman, G.L. (2001). A default mode of brain function. Proc. Natl. Acad. Sci. USA 98, 676-682.
11. Fox, M.D., Snyder, A.Z., Vincent, J.L., Corbetta, M., Van Essen, D.C., Raichle, M.E (2005). The human brain is intrinsically organized into dynamic, anticorrelated functional networks. Proc. Natl. Acad. Sci. USA 102, 9673-9678.
12. De Luca, M., Beckmann, C.F., De Stefano, N., Matthews, P.M., and Smith, S.M. (2006). fMRI resting state networks define distinct modes of long-distance interactions in the human brain. NeuroImage 29, 1359-1367.

Table S1. Fronto-Parietal Network

Region	Volume mm^3	% Region	Mean Z	Peak Z	Peak x	y	z
**Left Frontal Lobe**							
*Superior Frontal Gyrus	22156	76	5.09	12.74	-20	26	48
Medial Superior Frontal Gyrus	18168	76	4.30	11.68	-10	38	44
Orbital Superior Frontal Gyrus	2923	38	2.82	5.42	-31	55	-3
Middle Frontal Gyrus	27159	70	4.72	13.02	-22	26	48
Orbital Middle Frontal Gyrus	5954	83	3.98	8.67	-42	46	-8
Orbital Inferior Frontal Gyrus	6687	49	3.16	8.59	-42	46	-9
Triangular Inferior Frontal Gyrus	12047	59	2.33	6.46	-42	22	32
*Opercular Inferior Frontal Gyrus	4470	54	3.00	7.62	-42	21	36
Supplementary Motor Area	4763	27	2.49	9.20	-11	26	52
*Precentral Gyrus	7088	25	2.75	7.64	-42	12	44
**Left Parietal Lobe**							
*Angular Gyrus	9127	98	5.76	8.81	-46	-70	44
Inferior Parietal Lobule	9730	50	3.43	7.88	-50	-55	37
Supramarginal Gyrus	1571	15	2.23	5.92	-55	-53	32
Superior Parietal Lobule	2529	15	1.43	4.80	-37	-69	51
**Left Occipital & Temporal Lobes**							
Lateral Occipital Gyri	5729	22	2.89	7.74	-49	-70	39
Middle Temporal Gyrus	11955	30	2.53	7.06	-46	-62	24
Inferior Temporal Gyrus	7826	30	2.07	5.44	-54	-42	-16
**Right Frontal Lobe**							
Superior Frontal Gyrus	9413	29	3.20	7.04	18	30	44
Medial Superior Frontal Gyrus	5480	32	2.56	5.98	12	42	40
Middle Frontal Gyrus	8322	20	2.19	7.03	22	34	44
**Cerebellum**							
*Crus II	6504	38	2.07	4.94	38	-74	-44

Supplementary Table 1. *The fronto-parietal network of the test group, identified across both rest sessions using PICA.*

The fronto-parietal network (Figure 2, main paper) engaged the left parietal and frontal lobes, and to a lesser extent, the left temporal lobe, the right frontal lobe and the right cerebellum. The table lists the volume of the identified component within each anatomical region defined by the AAL atlas [9], the percent of the AAL region covered by the component, the mean z-score of the component within the AAL region, the peak z-score and the coordinates of the peak. Five coordinates chosen for whole-brain correlation analyses are indicated by asterisks*.

Table S2. Cerebellar Network

Region	Volume mm^3	% Region	Mean Z	Peak Z	Peak x	y	z
**Vermis**							
Lobule 1 & 2	383	95	2.83	7.41	6	-45	-22
Lobule 3	1608	88	3.97	8.64	2	-34	-12
Lobule 4 & 5	3647	69	4.20	12.32	6	-58	-18
Lobule 6	2956	100	9.10	13.42	6	-62	-20
Lobule 7	1564	100	9.26	12.68	2	-62	-24
Lobule 8	1940	100	9.36	12.34	-2	-62	-26
Lobule 9	1276	93	6.96	10.31	2	-59	-34
Lobule 10	675	77	3.11	9.53	2	-51	-24
**Left hemisphere**							
Lobule 3	985	92	3.07	8.15	-4	-47	-20
Lobule 4 & 5	6984	77	3.64	11.50	-4	-61	-17
Lobule 6	13108	96	6.44	12.13	-14	-66	-24
Crus1	14521	70	3.18	10.71	-14	-67	-26
Crus2	10696	70	2.88	9.26	-4	-68	-29
Lobule 7b	2919	63	2.84	8.12	-6	-71	-35
Lobule 8	10625	70	3.28	11.48	-4	-62	-29
Lobule 9	4946	71	2.45	8.39	-6	-56	-34
Lobule 10	719	62	1.42	6.34	-26	-41	-40
**Right hemisphere**							
Lobule 3	1212	76	2.77	8.90	7	-47	-20
Lobule 4 & 5	4857	72	3.25	12.63	8	-57	-20
Lobule 6	12996	90	6.50	13.60	10	-58	-20
Crus1	13452	64	2.97	9.82	14	-74	-25
Crus2	9383	55	2.05	10.42	5	-67	-28
Lobule 7b	2561	61	2.31	8.90	10	-72	-40
Lobule 8	13202	72	3.57	10.89	5	-63	-28
Lobule 9	4765	74	2.65	9.14	9	-56	-36
Lobule 10	528	41	1.06	5.57	26	-38	-40

Supplementary Table 2. *The cerebellar network for the test group across both rest sessions.* A single IC component covered much of the bilateral cerebellum (Figure 3, main paper). Above are the sub-volumes of the component within each anatomically (AAL) defined cerebellar region, the percent of the region covered by the component, the mean z-score within the region, the peak z-score within each region, and the location of the region's peak activation. This component was not identified in the analysis of the control group.

Table S3. Correlation Analysis

**Seed ROI**	**Volume mm^3**	**Peak Z**	**Peak location x**	**y**	**z**
**1 Superior frontal gyrus**					
Negative interaction					
L Fusiform cortex	40	3.413	-44	-18	-20
**2 Opercular inferior frontal gyrus**					
Positive interaction					
L Middle frontal gyrus	64	3.627	-40	28	34
Negative interaction					
L Cerebellum lobule IX	8	3.502	-6	-48	-52
**3 Precentral gyrus**					
Positive interaction					
L Middle frontal gyrus	144	3.989	-40	28	30
L Inferior frontal gyrus	72	3.402	-42	44	-8
Negative interaction					
L Cerebellum, crus I	8	3.14	-42	-70	-20
**4 Angular gyrus**					
Positive interaction					
L Inferior temporal gyrus	24	3.163	-56	-38	-16
Negative interaction					
L Hippocampus	128	3.63	-26	-28	-8
**5 Cerebellar Crus II**					
Positive interaction					
L Brainstem, Pons	8	3.150	-14	-30	-32

Supplementary Table 3. *The areas with significant group(test and control)×session (Rest 1 vs Rest 2) interaction in strength of correlation with regions of interest (ROIs) identified in Table 1.* The 2×2 ANOVA was used to find areas with significant interaction between group and session that demonstrate a learning-dependent change in correlation between the seed ROI and all other brain areas. For each of 5 seed ROIs, areas that were statistically significant for either the positive and negative interactions ($p=0.001$ uncorrected) are shown.

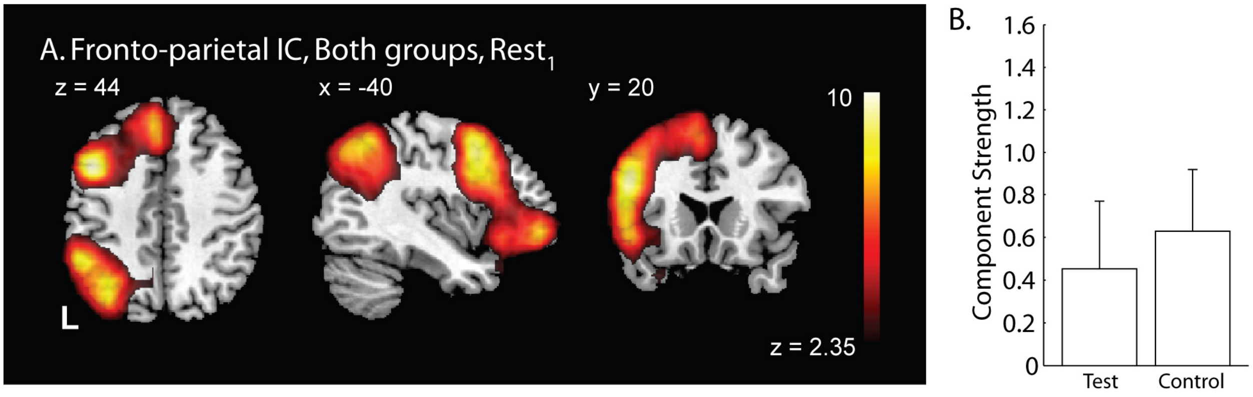

Figure S1. *The fronto-parietal component identified by PICA, which increased in strength following motor skill learning, is similar in strength prior to motor performance or motor learning in the test and control groups, respectively.* The component shown in panel A was reliable across the participants in both groups during the initial rest. The strength of the component did not vary between groups (panel B). This component includes the same areas as those in the initial analysis of the test group (see Supplementary Table 1), but includes a broader region within the right hemisphere of the cerebellum (not shown).

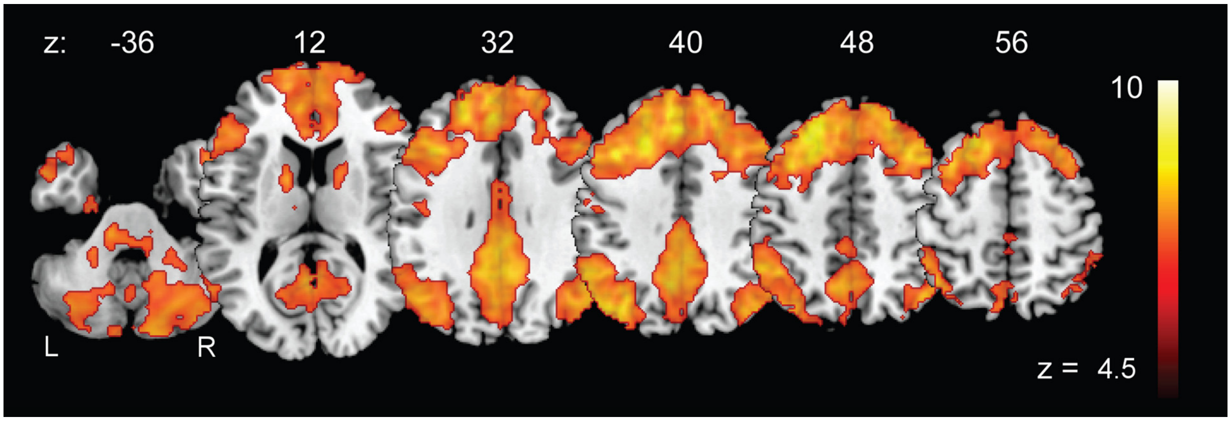

Figure S2. *Regions correlated with the left superior frontal gyrus seed.* The BOLD signal recorded during both sessions within the regions shown were significantly correlated with the activity in a seed region of interest centered on the superior frontal gyrus (Table 1, ROI 1), in both participant groups. The strength of the correlation did not significantly vary between groups. This component includes the same areas as those identified using PICA analysis (Supplementary Figure 1) but also includes a broader bilateral frontal region and a noticeable region within the medial parietal cortex, as frequently observed in default state analyses [10-12].

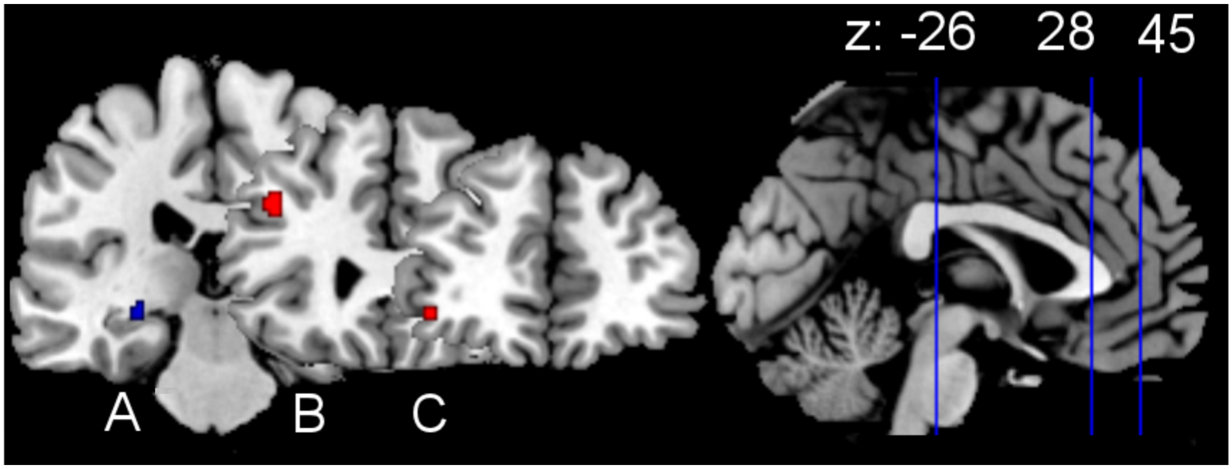

Figure S3. *Some of the areas significantly correlated with regions of interest centered on the precentral gyrus (red) or the angular gyrus (blue) (see Supplementary table 3, ROIs 3 and 4).* The regions were correlated with activity in the seed regions and the strength of the correlation was significantly increased (red) or decreased (blue) by learning – hence these areas showed a significant positive or negative group (test and control) ×session ($REST_1$ 1 vs $REST_2$) interaction in strength of correlation, respectively. The area in blue (A) is in the left hippocampus; red areas are in left middle (B: BA45) and inferior frontal gyri (C: BA47).
